# Reverse Phase High Performance Liquid Chromatographic Method for the Analysis of Letrozole in Pharmaceutical Dosage Forms

**DOI:** 10.4103/0250-474X.43019

**Published:** 2008

**Authors:** T. K. Laha, R. K. Patnaik, S. Sen

**Affiliations:** Cancer Chemotherapeutic Research Unit, College of Pharmaceutical Sciences, At/PO: Mohuda, Ganjam, Berhampur-760 002, India; 1 Kanaka Manjari Institute of Pharmaceutical Sciences, Rourkela-769 015, India

**Keywords:** RPHPLC, letrozole

## Abstract

A rapid and sensitive reverse phase high performance liquid chromatographic method is depicted for the qualitative and quantitative assay of letrozole in pharmaceutical dosage forms. Letrozole was chromatographed on a reverse phase C18 column with a mobile phase consisting of acetonitrile and phosphate buffer (pH 7.8) in the ratio of 70:30 v/v. The mobile phase was pumped at a flow rate of 1 ml/min. Acenaphthene was used as an internal standard and the eluents were monitored at 232 nm. The retention time of the drug was 3.385 min. With this method, linearity was observed in the range of 10-100 μg/ml. The LOD and LOQ were found to be 0.51 μg/ml and 1.52 μg/ml, respectively. The method was found to be applicable for analysis of drug in tablets. The results of the analysis were validated statistically.

Letrozole^1^ (benzonitrile, 4,4’-(1H-1,2,4-triazol-1-ylmethlene) bis-, Femara) is a potent and selective non-steroidal aromatase inhibitor, approved for use in post-menopausal women who have breast cancer that has progressed after antiestrogen therapy 1–5. A few methods of analysis of letrozole have been reported using different techniques such as microarray approach^6^, capillary gas chromatographic method with flame ionization detector^7^. However, there is no high performance liquid chromatographic (HPLC) method reported for letrozole in pharmaceutical dosage forms. The HPLC methods using the most commonly available columns and detector like UV are preferred. The present study describes the determination of letrozole in pharmaceutical dosage forms by using RP-C_18_ column with UV detectors. Owing to the widespread use of HPLC in routine analysis, it is important that well validated HPLC methods are to be developed for estimating letrozole. The aim of this study is development of a simple, precise, rapid and accurate reverse phase HPLC method for the estimation of letrozole in different pharmaceutical dosage forms.

The pure sample of letrozole used in this development of analytical method was gifted by Unimed Technologies Ltd, Baroda, India. Acetonitrile and Water were of HPLC grade (Merck India Ltd, Mumbai). All other reagents were of AR grade. An isocratic HPLC with a single Waters 510 Pump, Waters 486 tunable absorbance detector and RP-C_18_ column (Bondapak C_18_, 250×4.6 mm, packed with 5 μm particle size) was used. The HPLC system was equipped with Millennium32 software.

The mobile phase used was a mixture of acetonitrile and phosphate buffer (pH 7.8) in the ratio of 70:30 v/v; it was filtered before use through a 0.45 μm membrane filter and degassed for 30 min.The elution was carried out isocratically at the flow rate of 1.0 ml/min. Detection was carried out at 232 nm at ambient temperature.

Standard stock solution of letrozole (1 mg/ml) was prepared in mobile phase. To study the linearity range of the drugs, serial dilutions were made from standard stock solution in the range of 10-100 μg/ml. In each of them, 50 μg/ml acenaphthene was added as an internal standard. Twenty microliters of each solution was injected into the HPLC system to obtain the chromatogram. From these chromatograms, the area under the peaks of the drug and the internal standard were noted. Using these values, the mean ratio of peak area of the drug to that of the internal standard for each dilution was calculated. The regression of the drug concentration over these ratios was computed. The method follows the regression equation y = 0.0131x+0.0094 (where y is ratio of area under the curve of the drug to that of the internal standard and x is the corresponding concentration of letrozole) with a coefficient of correlation (r = 0.9993). The recovery studies were carried out by adding known amounts of letrozole to the preanalyzed samples and then analyzing them by the proposed HPLC method.

Twenty tablets were weighed and powdered. An accurately weighed portion of this powder equivalent to 50 mg of letrozole was transferred to a 50 ml volumetric flask containing 25 ml of the mobile phase. The contents of the flask were allowed to stand for 6 hours with intermittent sonication to ensure complete solubility of the drug. The mixture was made up to the volume with mobile phase, thoroughly mixed and then filtered through 0.45 μm membrane filter. From the filtrate, different aliquots were taken in separate 10 ml volumetric flasks. These solutions were spiked with suitable volume of the internal standard solution, such that the concentration of each solution was 50 μg/ml. The contents of the flasks was made up to the volume with the mobile phase and mixed well. Each of these solutions (20 μl) was then injected 5 times into the column. The mean peak area ratio of the drug to the internal standard of 5 such determinations was calculated and the drug content in the tablets was quantified using the regression equation obtained for the pure sample.

To achieve sharp peaks with good resolution, under isocratic conditions, mixtures of acetonitrole and phosphate buffer in different combinations were tested as mobile phase on a C_18_ stationary phase. A binary mixture of acetonitrile and phosphate buffer (pH 7.8) in 70:30 v/v proportions was proved to be the most suitable of all combination, since the chromatographic peaks were better defined, resolved and free from tailing with this system. Though the structure of acenaphthene is not similar to letrozole, it was chosen as an internal standard, because it showed better peak shape and peak location compared to other potential internal standards such as tadalafil and mirtazapine in this perspective. Under the above-mentioned chromatographic conditions, the retention time obtained for letrozole and the internal standard were 3.385 and 5.035 min, respectively (fig. 1). Each of the samples was injected 5 times and almost same retention times were observed in all cases. When letrozole solutions containing 50, 60 and 70 μg/ml were analyzed by the proposed method for finding out intra- and inter-day variations, a low coefficient of variation was observed (1.81%). This shows that the present HPLC method is highly precise. A recovery of 99.53% of letrozole from the preanalyzed samples shows that the present method is highly accurate.

**Fig. 1:**
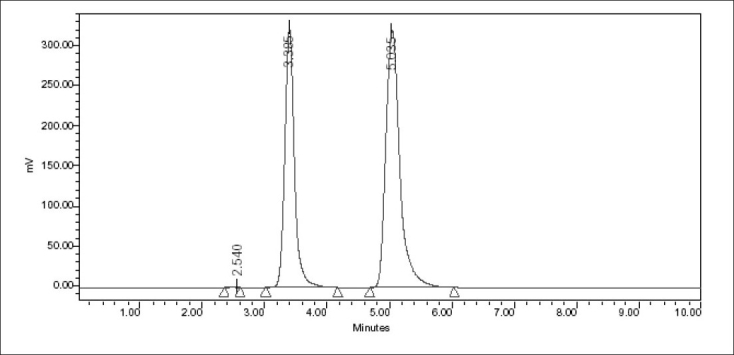
Typical chromatogram of letrozole and acenaphthene

The drug content in the tablets was quantified using the proposed analytical method. The mean amount of letrozole in two different brands of tablet dosage form is shown in Table 1. The absence of additional peaks in the chromatogram indicates non-interference of the common excipients used in the tablets. The tablets were found to contain 97.84 to 99.2% of the labeled amount of the drug. The low coefficient of variation indicates the reproducibility of the assay of letrozole in tablets. It can be concluded that the proposed HPLC method is sensitive and reproducible for the analysis of letrozole in pharmaceutical dosage forms within a short analysis time.

**TABLE 1:** RECOVERY STUDY

Drug	Assay results		Amount added μg/ml	Amount recovered μg/ml (n=5)	Recovery (%)	Average Recovery (%)
	
	Actual concentration (mg)	Concentration found (%) (n=5)				
Shantroz	2.5	97.84±2.61	16	15.69±0.43	98.09±2.73	98.42
			20	19.52±0.45	97.62±2.25	
			24	23.89±0.37	99.56±1.55	
Momazol	2.5	99.2±0.56	16	15.86±0.53	99.22±1.53	99.53
			20	19.89±1.01	99.34±0.96	
			24	24.01±0.63	100.03±0.77	

## References

[1] Barrows LR, Gennaro AR (2000). Antineoplastic and immunoactive drugs. Remington: The Science and Practice of Pharmacy.

[2] Lamb HM, Adkins JC (1998). Letrozole: A review of its use in postmenopausal women with advanced breast cancer. Drugs.

[3] Levien T, Baker DE (1997). Letrozole and Budesonide Inhalation powder. Hosp Pharma.

[4] Mouridsen H, Gershanovich M, Sun Y (2001). Superior efficacy of Letrozole versus Tamoxifen as first line therapy for postmenopausal women with advanced breast cancer. J Clin Oncol.

[5] Worthington I (1996). Current drug topics: Letrozole. Newsletter.

[6] Itoh T, Karlsberg K, Kijima I, Yuan YC, Smith D (2005). Letrozole: A microarray Approach. Mol Cancer Res.

[7] Berzas JJ, Rodriguez J, Contento AM, Cabello MP (2003). Determination of drugs used in advanced breast cancer by capillary gas chromatography of pharmaceutical formulations. J Separation Sci.

